# An update on the diagnosis of growth hormone deficiency

**DOI:** 10.15190/d.2018.2

**Published:** 2018-04-12

**Authors:** Georgiana Roxana Gabreanu

**Affiliations:** Carol Davila University of Medicine and Pharmacy, Bucharest, Romania

**Keywords:** Growth hormone deficiency, Insulin-like Growth Factor-1, IGF- Binding protein 3, insuline tolerance test (ITT), GHRH-arginine stimulation test, Glucagon stimulation test, macimorelin, genetic testing

## Abstract

Growth hormone deficiency (GHD) is an endocrine disorder, which may be either isolated or associated with other pituitary hormone deficiencies. In children, short stature is a useful clinical marker for GHD. In contrast, symptomatology is not always so obvious in adults, and the existing methods of testing might be inaccurate and imprecise, especially in the lack of a suggestive clinical profile. Since the quality of life of patients diagnosed with GHD could also be significantly affected, in both children and adults, a correct and accurate diagnosis is therefore tremendously important to select those patients that can benefit from the GH treatment. In general, the endocrine diseases are challenging in terms of diagnosis, the simple measurement of the basal level of hormones is not sufficient for distinguishing between the physiological and pathological conditions. Traditionally, several stimulation tests have been considered by professional clinical guidelines, such as insulin tolerance test (ITT), GHRH-arginine stimulation test and the glucagon stimulation test, and all of them have both advantages and limitations. More recently (December 2017), FDA approved a growth hormone secretagogue receptor agonist, macimorelin, for the diagnosis of adults with GHD. The obvious advantage for macimorelin is the simple oral administration and the high level of agreement with the insulin tolerance test for those patients with organic disease and low levels of insulin-like growth factor (IGF-I). However, the safety profile and the diagnostic value was not yet established for the pediatric population and for those adults with extreme or morbid obesity. In addition, administration of macimorelin with drugs that prolong QT interval and CYP3A4 inducers should be avoided. Genetic screening could obviously bring a great insight in the GHD pathology. However, it remains an open question if it would be also cost effective to include it in the routine evaluation of the patients with GHD. Although major progresses have been made in this area, genetic testing continues to be difficult to access, mostly because of its high costs, especially in the low-income and middle-income countries.

## 1. Introduction

**Figure 1:**
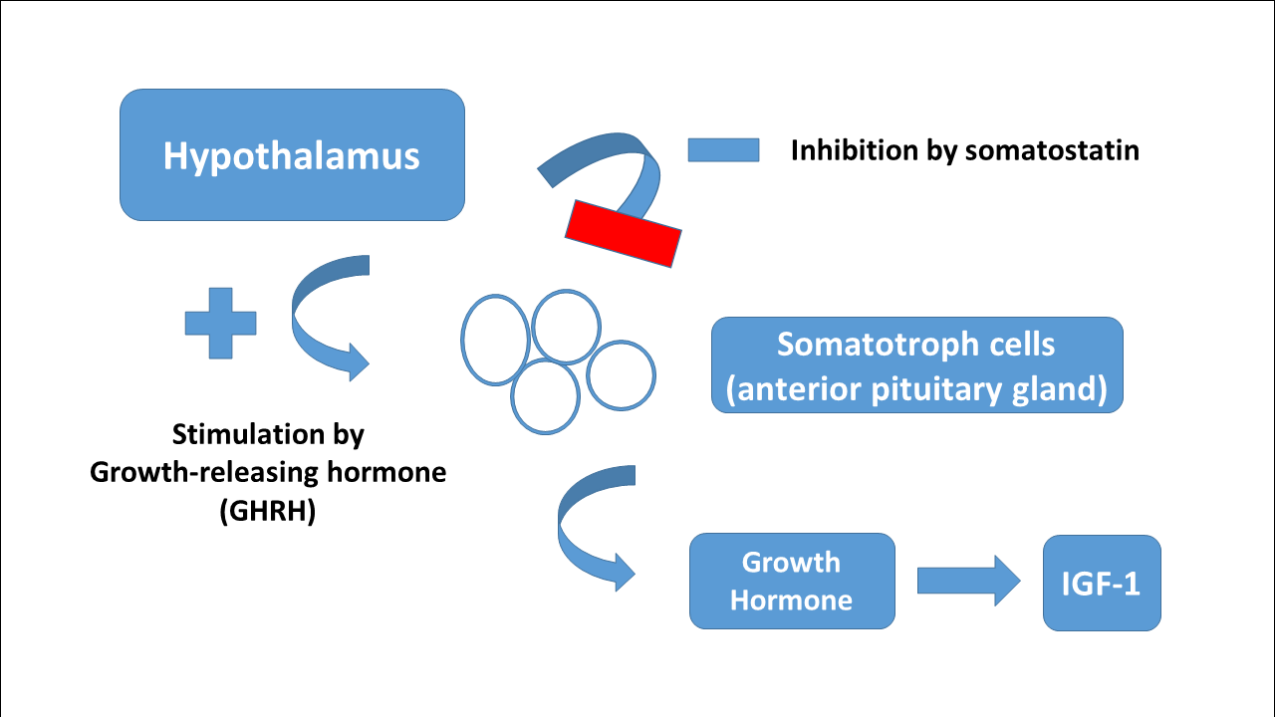
Regulation of Growth Hormone Secretion

Growth hormone is a polypeptide secreted by the somatotropic cells of the anterior pituitary, and the process is regulated by complex mediators and mechanisms. Some of the key regulatory factors are GH releasing hormone (GHRH), somatostatin, GH releasing peptide and insulin-like growth factor (IGF-I) (**Figure 1**). The deficiency of growth hormone is an endocrine disorder that may be isolated or associated with other pituitary hormone deficiencies^1^.

Growth hormone deficiency occurs in both children and adults. As the Endocrine Society indicates, childhood-onset GHD may be triggered by organic causes, or it can be idiopathic, with an unknown cause^2^**. **Patients with childhood-onset GHD are recommended to be retested as adults, since cases of reconversion to a normal GH response were described, especially in the idiopathic GHD (the cause is unknown)^2,3^. However, it is not necessary to reassess those patients with mutations or irreversible lesions, as they do not revert to normal GH responses^2^. Thus, a proportion of the adults with GHD diagnose is represented by those with prior childhood-onset GHD. Another proportion of adults with GHD is represented by those with acquired deficiency during adulthood. A clinical practice guideline on adult growth hormone deficiency was established in 2011 by the Endocrine Society. The adult patients that are recommended by the authors to be tested for growth hormone deficiency are those who experienced surgical interventions or irradiation due to several brain tumors or any other pathology that could affect the hypothalamus or the pituitary gland, and subsequently would lead to acquired GHD. Finally, although rare, idiopathic GHD could also occur in adults^2^.

While in children short stature is a useful clinical marker for GHD, symptomatology is not always so obvious in adults, consisting of nonspecific clinical signs, such as reduced muscle mass with fatigue and poor exercise capacity, glucose intolerance, atherosclerosis and osteoporosis^2,4^. The Endocrine Society recommends testing only for patients with a probability to be diagnosed with GHD based on medical history and clinical symptomatology. Otherwise, the existing methods of testing might be inaccurate and imprecise^2^. Therefore, those patients lacking a suggestive clinical profile (especially the idiopathic GHD ones) are recommended to be tested with two stimulation tests for the final diagnosis, since they show a high rate of false-positive in response to a single GH stimulation test^2^.

In addition, the quality of life of patients diagnosed with growth hormone deficiency could be significantly affected, in both children and adults. A very interesting report based on interviews with adult patients diagnosed with GHD highlights a mixture of feelings. Some of the patients feel “embarrassed” of their body image, they have a sense of ”failure”, and they sometimes end up in depression, stress and anxiety. The social life could be also affected, and this is sometimes due to the lack of energy and persistent fatigue to meet friends or to continue certain projects. Also, irregular sleeping patterns worsen the fatigue in some of the patients, others experience memory loss or they “struggle” to focus or concentrate on daily tasks, such as reading “a paragraph in a magazine article”^5^. A correct and accurate diagnosis is therefore tremendously important to select those patients that can benefit from the GH treatment.

## 2. Growth Hormone and Insulin-like Growth Factor-1 (IGF-1))

**Majority of endocrine diseases are challenging in terms of diagnosis and the simple measurement of the basal level of hormones is not sufficient for distinguishing between the physiological and pathological conditions^6^. The growth hormone is secreted from the anterior pituitary in approximately 10 pulses per day, which means that a single measurement of GH could not be of diagnostic value, since the level of GH is only detectable about 90 minutes per day in basal conditions^7^. Thus, Auernhammer et al. highlight in a recent paper the remarkable role of the functional diagnosis in endocrinology, which is represented by stimulation tests when the evaluation of the growth hormone deficiency is needed^6^. Growth hormone stimulates the production of insulin-like growth factor (IGF) from liver, muscle and bone. More than 75% of IGF1 binds with high affinity to the IGF binding protein 3 (IGFBP3), and this complex is subsequently stabilized by Acid Labile Subunit (ALS), which leads to an increased half-life of 16 hours. These two molecules could be used in the evaluation of GH status, however they could be biased by several external factors, such as the nutritional status, age, thyroid hormones and hepatic function^7^. The Growth Hormone Research Society strongly recommends in a consensus statement on the diagnosis of children with idiopathic short stature (2008) that IGF1 levels should be measured as part of evaluation of children with low height velocity^8^. According to the Endocrine Society’s clinical guideline, the low level of IGF-I after discontinuation of GH treatment for at least 1 month is sufficient to confirm the diagnostic of GHD in those children with irreversible lesions or documented genetic mutations, and no additional stimulation tests are required^2^. Low levels of IGF1 and IGFBP3 have been shown to have high specificity for GHD, especially for young children, and even higher when combined with height velocity in the pediatric population. However, normal values cannot exclude GHD and should be confirmed with other tests, since the sensitivity is relatively low^7^. Differences of IGF1 levels may arise between various immunoassays, thus it is recommended to use the same type of IGF assay throughout the entire follow-up of a patient, to minimize the bias^9^. In 2011, an international consensus on behalf of the Growth Hormone Research Society, the IGF society, the Pituitary Society and the IFCC was published regarding the standardization and evaluation of growth hormone and insulin-like growth factor assays. In order to improve assay comparison, it is recommended to use an international standard for each hormone assay, to implement internal quality control programs and to participate in an accredited proficiency testing/external quality assessment program^10^.

## 3. Stimulation tests for growth hormone deficiency (GHD) diagnosis

### Conventional stimulation tests for adults

Several stimulation tests have been described for adult GHD diagnosis, including insulin tolerance test, stimulation with growth-hormone releasing hormone - arginine, stimulation with glucagon, arginine and macimorelin (**Table 1** - GH stimulation tests in adult GHD)^11-13^.

Traditionally, insulin tolerance test is largely considered the ”gold standard’’ test for GHD in adults and it is recommended by the Endocrine Society for its sensitivity and specificity for diagnosing adult GHD, although it should be avoided in patients with medical history of seizures and cardiovascular pathologies^2^. When contraindications for the insulin tolerance test exist, the Endocrine Society recommends as an alternative test the combined administration of GH releasing hormone and arginine (GHRH-arginine), although not always available^2^. In 1998, the consensus guideline elaborated by the Growth Hormone Research Society, from a variety of stimulation tests that had been used, considered only two tests of well validated diagnostic value, namely ITT and GHRH-arginine^14^. Despite these recommendations, a later study performed in 2002 in USA, showed that, from 817 patients evaluated for GHD, only 11.4% of them underwent an insulin tolerance test and less than 1% of patients were tested with arginine combined with GHRH. In contrast, 61 % of patients were tested with arginine alone or L-Dopa^15^. Later, in 2007, the Growth Hormone Research Society published an update on diagnosis in adult GHD, emphasizing that several stimulation tests gained diagnostic value and have been validated since the last consensus guideline. Thus, GHRH plus arginine, GHRH plus growth hormone-releasing peptide (GHRP), and glucagon stimulation tests gained popularity among endocrinologists for the purpose of diagnosing GHD^16^. Shortly after, in 2008, GHRH became unavailable in the USA, determining the guidelines to consider a second alternative for the patients with ITT contraindications. Thus, the Endocrine Society and the American Association of Clinical Endocrinologists now recommend using the glucagon stimulation test (GST) for GHD diagnose in adult patients with contraindications for ITT, and when GHRH is unavailable^2,17^.

### Conventional stimulation tests for children

Although insulin tolerance test have been considered the ,,gold standard’’ test for GHD in adults, there is no consensus for the first test choice for GHD in children. Interestingly, a recent study published in 2017 shows a high rate of false positive associated with the insulin tolerance test when used as a first test, and it suggests arginine test to be a better option in children^18^.

Several stimulation tests have been used for GHD diagnosis in children, including insulin tolerance test, stimulation with clonidine, glucagon, levodopa, arginine and growth-hormone releasing hormone (**Table 2** - GH stimulation tests in GHD in children). While low levels of GH after stimulation tests are reported in general for severe GHD, there is no clear threshold for distinguishing between mild GHD and healthy controls, as highlighted in the guidelines on GHD of the Pediatric Endocrine Society (2016). The cut-offs for GH values in the pediatric population are also highly debated, and several peak GH levels have been considered to indicate GHD (below 7 or 10 µg/L)^7^.

### Recently validated stimulation test, macimorelin, for adult GHD diagnosis

Recently, a growth hormone secretagogue receptor agonist, macimorelin, was approved by FDA for the diagnosis of adults with GHD *(Macrilen, Aeterna Zentaris, Approved: December 2017)*^19^.

Simple administration in a single oral dose of 0.5 mg/kg is an obvious advantage. Firstly, the healthcare professional reconstitutes a solution using one pouch of macimorelin (60 mg) for every 120 mL of water, resulting a solution of 0.5 mg/mL. Based on the patient weight, the appropriate volume of macimorelin solution is orally administered and the dynamics of GH is measured in four time points,at 30 minutes, 45 minutes, 60 minutes and 90 minutes. After stimulation, the adult growth hormone deficiency can be confirmed if the maximum of serum GH level is less than 2.8 ng/m^20^.

Macimorelin stimulation test was compared with ITT in a randomized, open-label, cross-over study, in order to establish the diagnostic efficacy of macimorelin. Patients were classified into three groups, according to the likelihood of being diagnosed with GHD, and one group of healthy adult controls. The best level of negative and positive agreement between the results of the ITT and macimorelin were obtained for the group A, those with a high likelihood of GHD (positive agreement 89% and negative agreement 100%). These patients have in common low insulin-like growth factor 1 (IGF-1) and structural damage at the hypothalamus or pituitary level with childhood onset or acquired during adulthood and/or three or more pituitary hormone deficiencies. This is in accordance with the GHD guidelines which state that the accuracy of a stimulation test increases when combined with organic disease and low levels of IGF-1^20^.

Several of its limitations are linked to the fact that the safety profile and the diagnostic value was not established yet for the pediatric population and for those adult patients with body mass index more than 40 kg/m^2^, also classified as extreme or morbid obesity (class III obesity)^20,21^. Regarding the safety profile in adult patients, some of the most common adverse effects were the distortion of the sense of taste, dizziness, headache, fatigue and gastrointestinal reactions (nausea, hunger and diarrhea). Also, administration with drugs that prolong QT interval (antipsychotic drugs, some antibiotics and some of the antiarrhythmic medications) should be avoided, since it may lead to ventricular tachycardia. Additionally, since CYP3A4 is the major enzyme to metabolize macimorelin, false positives could be obtained if used with cytochrome P450 (CYP) 3A4 inducers, due to the fact that macimorelin level is reduced and it, therefore, fails to stimulate GH^20^.

**Table 1:** GH stimulation tests: GHD diagnosis in ADULTS

GHD stimulation test	GH measurements	Administration	Observations	Reference
Insulin tolerance test	7 GH measurements between 20-90 min after hypoglycemia is achieved (<40 mg/dL)	Intravenous	Considered the "gold standard". It should be avoided in patients with medical history of seizures and cardiovascular pathologies	^2,11-13^
GHRH-arginine	4 GH measurements between 30-120 min	Intravenous	Became unavailable in the US in 2008	^11-13^
Glucagon stimulation test	9 GH measurements between 0-240 min	Intramuscular	Nausea and late hypoglycemia	^11-13^
Arginine alone	4 GH measurements between 30-120 min	Intravenous	Nausea, headache, vomiting	^11-13^
Macimorelin	4 GH measurements between 30-90 min	Oral admin. in a single dose	Safety profile & the diagnostic value was not yet established for patients with body mass index > 40 kg/m^2^ & for the pediatric population	^20^

**Table 2:** GH stimulation tests: GHD diagnosis in CHILDRENS

GHD stimulation test	GH measurements	Administration	Observations	Reference
Insulin tolerance test	5 GH measurements between 0-120 min	Intravenous	Associated with a high rate of false positive when used as a first test; risk of hypoglycemia	^6,8^
Clonidine	4 GH measurements between 0-90 min	Oral	Hypotension and drowsiness	^6^
Glucagon	5 GH measurements between 0-3 h	Intramuscular		^6^
Levodopa	5 GH measurements between 0-120 min	Subcutaneous/ Intramuscular	Vomiting and headache	^6^
Arginine	5 GH measurements between 0-120 min	Intravenous	Suggested to be a better option compared with ITT (lower rate of false positives when used as a first test). Not widely available	^6,8^
GHRH	5 GH measurements between 0-60 min	Intravenous	Not widely available	^6^

## 4. Proteomics in growth hormone deficiency

Only a few studies have reported potential protein biomarkers in the diagnosis of GHD. A recent article published in 2018, based on next-generation proteomics SWATH-MS technology, reported three proteins that could be useful as simple, non-invasive, cost-effective biomarkers for GHD, namely apolipoprotein A-IV, complement factor H-related protein 4 and platelet basic protein. The study investigated a total of 263 proteins in 15 children with GHD compared to 15 healthy controls^22^. In addition, potential biomarkers for GH treatment monitoring were described. Five isoforms of haptoglobin and one isoform of apolipoprotein A-I were significantly modified after the GH treatment^23^.

## 5. Genetic screening

Somatotropic cells of the anterior pituitary gland express the human growth hormone gene, which resides on the chromosome 17q22-24^24^. Between 3-30% of the cases with GHD are suggested to have a genetic cause, and the percent it might be even higher, since imagistic methods, such as magnetic resonance, roughly detect 12-20% of the lesions of the hypothalamus and pituitary gland. At least four Mendelian disorders of familial isolated growth hormone deficiency have been described (type IA, IB, which are autosomal recessive, type II, which is autosomal dominant, and type III, which is X-linked)^25^.

Numerous mutations in the GH gene and GHRH receptor gene have been reported in patients with congenital isolated GHD^26^. A recent study published in February 2018 describes a novel mutation (c.97C>T) of the GH releasing hormone receptor gene that causes isolated GHD type IB. The variant was identified at the evaluation of a pediatric patient with severe growth failure, who expressed low GH in response to 2 stimulation tests^27^. Wit et al. recommends genetic screening for GH and GHRHR mutations in children with severe isolated GHD and a family history of GHD^28^.

Other genetic causes incriminated to have a role in the GHD pathology are the mutations in the Gs alpha gene leading to GHRH resistance and mutations in the gene encoding the growth hormone (GH) secretagogue receptor (GHSR)^29,30^. Also, a wide variety of mutations in genes encoding transcription factors involved in pituitary development were reported, such as: HESX1, OTX2, SOX2, SOX3, LHX3, PITX2, PROP1, POU1F1 and TCF7L1. Additionally, two KCNQ1 mutations have been recently described to be involved in growth hormone deficiency pathology^31^.

Genetic screening could obviously bring a great insight in the GHD pathology, but it remains an open question if it would be also cost effective to include it in the routine evaluation of the patients with GHD. Although major progress has been made in this area, genetic testing continues to be difficult to access, mostly because of its high costs, especially in the low-income and middle-income countries^32^.

## 6. Conclusion

There is a tremendous necessity for a correct and accurate diagnosis of growth hormone deficiency in order to select those patients that can benefit from the GH treatment. Diagnosis of GHD, as in many other endocrine disorders, remains a true challenge, because of the pulsatile secretion of hormones. Existing methods of testing might be inaccurate and imprecise, especially in the lack of a suggestive clinical profile. Moreover, the lack of GH and IGF1 assay standardization could be a source of bias, and it should be taken into ccount in the follow-up of a GHD patient. Although major progress has been made in the understanding of the genetic causes of the GHD, genetic screening continues to be difficult to access, mostly because of its high costs, especially in the low-income and middle-income countries. It remains to be elucidated if it is cost effective to be included in the routine evaluation of the patients with GHD.

## KEY POINTS

**◊ Several stimulation tests have been considered by professional clinical guidelines, including insulin tolerance test, GHRH-arginine and Glucagon stimulation tests.**

**◊ Recently, a growth hormone secretagogue receptor agonist, macimorelin, was approved by FDA (Dec. 2017) for the diagnosis of adults with GHD.**

**◊ A wide variety of genetic mutations have been reported to be involved in the GHD pathology.**

## OPEN QUESTION

**◊ Is genetic screening cost effective enough to be included within a routine evaluation of the worldwide patients with suspected GHD?**
